# Expansion Microscopy of Synaptic Contacts on the Mauthner Cells of Larval Zebrafish

**DOI:** 10.21769/BioProtoc.5067

**Published:** 2024-09-20

**Authors:** Sundas Ijaz, Sandra P. Cárdenas-García, Alberto E. Pereda

**Affiliations:** Dominick P. Purpura Department of Neuroscience, Albert Einstein College of Medicine, Bronx, NY, USA

**Keywords:** Auditory, Escape, Immunohistochemistry, Nanoscale imaging, Connexin, Electrical synapse

## Abstract

Because of its genetic tractability and amenability for live imaging, larval zebrafish (*Danio rerio*) have emerged as a model to study the cellular and synaptic properties underlying behavior. The accessibility of Mauthner cells, a pair of escape-organizing neurons located in the brainstem of teleost fish, along with their associated sensory inputs, enables exploration of the correlation between structural and functional synaptic features. This is the case of the endings of auditory afferents on the lateral dendrite of this cell, known as large myelinated club endings, which provide the excitatory drive for the initiation of auditory-evoked escape responses mediated by the Mauthner cell and its spinal network. Here, we describe the procedures that make it possible to expose the molecular composition of these synapses using protein-retention expansion microscopy (proExM). This method allowed us to generate a map of the distribution of synaptic proteins at these identifiable synapses, which could also be applied to examine the organization of other synaptic contacts in this cell.

Key features

• This protocol builds upon the method developed by Tillberg et al. [1]

• Optimized for the examination of the organization of molecular components at synaptic contacts on the Mauthner cells of larval zebrafish

• Requires at least three days to complete and should be preceded by immunostaining.

• Results in a linear expansion factor of ~3.9× and an area expansion factor of ~13×

## Background

The Mauthner cells are a pair of large reticulospinal neurons involved in tail-flip escape responses in fish [2,3]. Because of their experimental accessibility, these cells are considered a valuable model for studying vertebrate synaptic transmission, as they more easily allow for the correlation between the structure and function of synaptic features [4–7]. Auditory afferents originate in the sacculus, an organ of the vestibular system with auditory function in fish, and terminate as single large myelinated club endings [8,9] or club endings (CEs) on the lateral dendrite of the Mauthner cells. These terminals support both electrical and chemical synaptic transmission [5,10]. Because of their anatomical and physiological identifiability, these terminals have historically been amenable to exploring synaptic structure and function with novel technical approaches. For example, the seminal work by J.D. Robertson [4], using an electron microscope at these contacts, provided early evidence for the anatomical bases for electrical transmission at the gap junction. We have recently applied protein-retention expansion microscopy (proExM) to study the overall organization of these terminals. Unlike the more labor-intensive electron microscopy, this technique allowed us to generate a map of the incidence and distribution of synaptic proteins associated with electrical and glutamatergic transmission [11]. ProExM was able to expose, with sufficient resolution, spatial features that have only been observed so far with electron microscopy. Moreover, unlike electron microscopy, expansion microscopy allowed us to more easily generate a map of the organization of the synaptic contact by labeling proteins that form its various synaptic components. The protocol that we describe here is based on a previously established methodology [1]; we propose that it would be useful for exploring the anatomical organization of other synapses on the distal portion of the Mauthner cell lateral dendrite. This includes inhibitory small vesicle boutons (SVBs) [12], known to surround CEs, as well as synapses located in other processes of this large cell and throughout the zebrafish brain in general.

## Materials and reagents

**Reagents**

1. 10× phosphate-buffered saline (PBS) (Sigma-Aldrich, catalog number: 6506-OP)
2. Dimethyl sulfoxide (DMSO) (Honeywell, catalog number: 67-68-5)
3. Cell culture–grade water (Sigma-Aldrich, catalog number: W3500)
4. Trichloroacetic acid (TCA) (Sigma-Aldrich, catalog number: T6399)
5. Normal goat serum (NGS) (Vector Laboratories, catalog number: S-1000)
6. Acryloyl-X, SE (AcX) (Invitrogen, catalog number: A20770)
7. Primary antibodies used for immunostaining:
  - Mouse IgG1 anti-Cx35/36 (monoclonal) (Millipore Sigma, catalog number: MAB3045, 1:250 dilution)
  - Rabbit anti-Cx35.5 (monoclonal) (Fred Hutch Antibody Technology Facility: clone12H5, 1:200 dilution)
  - Mouse IgG2A anti-Cx34.1 (monoclonal) (Fred Hutch Antibody Technology Facility: clone5C10A, 1:200 dilution)
  - Mouse IgG1 anti-ZO1 (monoclonal) (Invitrogen, catalog number: 33-9100, 1:200 dilution)
  - Mouse IgG1 anti-N-cadherin (monoclonal) (BD Transduction Laboratories, catalog number: 610920, 1:50 dilution)
  - Mouse IgG1 anti-Beta-catenin (monoclonal) (Sigma, catalog number: C7207, 1:100 dilution)
  - Chicken IgY anti-GFP (polyclonal) (Abcam, catalog number: ab13970, 1:200 dilution)
  - Rabbit IgG anti-GluR2/3 (polyclonal) (EMD Millipore, catalog number: 07-598, 1:200 dilution)
8. Secondary antibodies used for immunostaining:
  - Mouse IgG Alexa Fluor 546 (polyclonal) (Invitrogen, catalog number: A11030, 1:200 dilution)
  - Mouse IgG Atto 647N (polyclonal) (Sigma-Aldrich, catalog number: 50185, 1:200 dilution)
  - Rabbit IgG Alexa fluor 546 (polyclonal) (Invitrogen, catalog number: A11010, 1:200 dilution)
  - Rabbit IgG Atto 647N (polyclonal) (Sigma-Aldrich, catalog number: 40839, 1:200 dilution)
  - Chicken IgY Alexa Fluor 488 (polyclonal) (Invitrogen, catalog number: A11039, 1:200 dilution)
9. Sodium acrylate (Sigma-Aldrich, catalog number: 408220)
10. Acrylamide (Sigma-Aldrich, catalog number: A9099)
11. N,N'-methylenebisacrylamide (Sigma-Aldrich, catalog number: M7279)
12. Sodium chloride (NaCl) (Sigma-Aldrich, catalog number: S9888)
13. 4-hydroxy-TEMPO (4-HT) (Sigma-Aldrich, catalog number: 176141)
14. Tetramethylethylenediamine (TEMED) (Sigma-Aldrich, catalog number: T7024)
15. Ammonium persulfate (APS) (Sigma-Aldrich, catalog number: A3678)
16. Triton X-100 (Sigma-Aldrich, catalog number: X100)
17. 1 M tris(hydroxymethyl)aminomethane (Tris), pH 8.0 (Invitrogen, catalog number: AM9855G)
18. 0.5 M Ethylenediaminetetraacetic acid (EDTA) (Invitrogen, catalog number: AM9260G)
19. Proteinase K, >600 U/mL (Thermo Fisher, catalog number: EO0491)
20. Poly-D-Lysine, 1 mg/mL (Sigma-Aldrich, catalog number: A-003-E)
21. Reagent alcohol (ethanol) (Sigma-Aldrich, catalog number: 362808)

**Solutions**

1. 10% blocking solution (see Recipes)
2. 2% trichloroacetic acid (TCA) solution (see Recipes)
3. 1% acryloyl-X, SE (AcX) stock solution (see Recipes)
4. 38% sodium acrylate stock solution (see Recipes)
5. 50% acrylamide stock solution (see Recipes)
6. 2% N,N'-methylenebisacrylamide stock solution (see Recipes)
7. 5 M sodium chloride (NaCl) stock solution (see Recipes)
8. 0.5% 4-hydroxy-TEMPO (4-HT) stock solution (see Recipes)
9. 10% tetramethylethylenediamine (TEMED) stock solution (see Recipes)
10. 10% ammonium persulfate (APS) stock solution (see Recipes)
11. Anchoring solution (see Recipes)
12. Monomer solution (see Recipes)
13. Gelling solution (see Recipes)
14. Digestion buffer (see Recipes)
15. Proteinase K solution (8 U/mL) (see Recipes)
16. 70% ethanol (see Recipes)

**Recipes**

1. **10% blocking solution**
*Note: Prepare this fresh with each experiment.*
  **Table:** 

Reagent	Final concentration	Quantity or Volume
NGS	10% (v/v)	200 μL
DMSO	1% (v/v)	20 μL
0.5% Triton X-100 in PBS (PBS-Trx)	n/a	Fill to a final volume of 2 mL
2. **2% trichloroacetic acid (TCA) solution**
*Note: Prepare this fresh with each experiment.*
  **Table:** 

Reagent	Final concentration	Quantity or Volume
TCA	2% (w/v)	1 g
1× PBS	n/a	Fill to a final volume of 50 mL
3. **1% acryloyl-X, SE (AcX) stock solution**
*Note: Prepare 20 μL aliquots of this and store at -20 °C with a drying agent for up to two months.*
  **Table:** 

Reagent	Final concentration	Quantity or Volume
AcX	1% (w/v)	5 mg
DMSO	n/a	500 μL
4. **38% sodium acrylate stock solution**
*Note: Store this at 4 °C for up to one month.*
  **Table:** 

Reagent	Final concentration	Quantity or Volume
Sodium acrylate	38% (w/v)	1.9 g
Cell culture–grade water	n/a	Fill to a final volume of 5 mL
5. **50% acrylamide stock solution**
*Note: Store this at 4 °C for up to six months.*
  **Table:** 

Reagent	Final concentration	Quantity or Volume
Acrylamide	50% (w/v)	5 g
Cell culture–grade water	n/a	Fill to a final volume of 10 mL
6. **2% N,N'-methylenebisacrylamide stock solution**
*Note: Store this at 4 °C for up to six months.*
  **Table:** 

Reagent	Final concentration	Quantity or Volume
N,N'-methylenebisacrylamide	2% (w/v)	0.2 g
Cell culture–grade water	n/a	Fill to a final volume of 10 mL
7. **5 M Sodium chloride (NaCl) stock solution**
  **Table:** 

Reagent	Final concentration	Quantity or Volume
NaCl	5 M	14.6 g
Cell culture–grade water	n/a	Fill to a final volume of 50 mL
8. **0.5% 4-hydroxy-TEMPO (4-HT) stock solution**
*Note: Prepare 100 μL aliquots of this and store at -20 °C with a drying agent for up to one month.*
  **Table:** 

Reagent	Final concentration	Quantity or Volume
4-HT	0.5% (w/v)	50 mg
Cell culture–grade water	n/a	Fill to a final volume of 10 mL
9. **10% Tetramethylethylenediamine (TEMED) stock solution**
*Note: Prepare 100 μL aliquots of this and store at -20 °C with a drying agent for up to one month.*
  **Table:** 

Reagent	Final concentration	Quantity or Volume
TEMED	10% (v/v)	20 μL
Cell culture–grade water	n/a	180 μL
Total	n/a	200 μL
10. **10% ammonium persulfate (APS) stock solution**
*Note: Prepare 100 μL aliquots of this and store at -20 °C with a drying agent for up to one month.*
  **Table:** 

Reagent	Final concentration	Quantity or Volume
APS	10% (w/v)	1 g
Cell culture–grade water	n/a	Fill to a final volume of 10 mL
11. **Anchoring solution**
  **Table:** 

Reagent	Final concentration	Quantity or Volume
1% AcX stock solution	0.01% (v/v)	2 μL (dilute 1:100)
1× PBS	n/a	198 μL
Total	n/a	200 μL
12. **Monomer solution**
*Note: The final concentration of these reagents will decrease slightly in the gelling solution with the addition of 4-HT, TEMED, and APS. These concentrations are listed in parenthesis. Additionally, this solution should be made fresh with every experiment.*
  **Table:** 

Reagent	Final concentration	Quantity or Volume
38% sodium acrylate stock solution	9.1% (8.6%)	2.25 mL
50% acrylamide stock solution	2.7% (2.5%)	0.5 mL
2% N,N'-methylenebisacrylamide stock solution	0.16% (0.15%)	0.75 mL
5 M NaCl stock solution	2.13 M (2 M)	4 mL
10× PBS	1.06× (1×)	1 mL
Cell culture–grade water	n/a	0.9 mL
Total	n/a	9.4 mL
13. **Gelling solution**
*Note: 4-HT, TEMED, and APS should be added sequentially, in the order they are listed, per individual sample. This solution should not be mixed beforehand.*
  **Table:** 

Reagent	Final concentration	Quantity or Volume
Monomer solution	n/a	376 μL
0.5% 4-HT stock solution	0.01%	8 μL
10% TEMED stock solution	0.2%	8 μL
10% APS stock solution	0.2%	8 μL
Total	n/a	400 μL
14. **Digestion buffer**
*Note: Prepare 5 mL aliquots of this and store at -20 °C for up to one year.*
  **Table:** 

Reagent	Final concentration	Quantity or Volume
1 M Tris (pH 8.0)	50 mM	2.5 mL
0.5 M EDTA	1 mM	0.1 mL
Triton X-100	0.5% (v/v)	0.25 mL
5 M NaCl stock solution	0.5 M	5 mL
Cell culture–grade water	n/a	42.15 mL
Total	n/a	50 mL
15. **Proteinase K solution (8 U/mL)**
*Note: Proteinase K should only be added prior to the digestion step. Do not freeze with digestion buffer for storage.*
  **Table:** 

Reagent	Final concentration	Quantity or Volume
Proteinase K, >600 U/mL	8 U/mL	3.3 μL
Digestion buffer	n/a	246.7 μL
Total	n/a	250 μL
16. **70% ethanol**
  **Table:** 

Reagent	Final concentration	Quantity or Volume
Ethanol	70%	35 mL
Cell culture–grade water	n/a	15 mL
Total	n/a	50 mL

**Laboratory supplies**

1. Fine Science Tools Dumont #4 forceps 0.13 × 0.08 mm, 11 cm (Fisher Scientific, catalog number: NC9091939)
2. Fisherbrand Premium plain microscope slides, 25 × 75 × 1.0 mm (Fisher Scientific, catalog number: 125444)
3. Epredia cover slips, 22 × 22, No.1 (Fisher Scientific, catalog number: 102222)
4. Fisherbrand cover slips, 24 × 50, No.1 (Fisher Scientific, catalog number: 12-545-88)
5. Falcon 24-well tissue culture plate (Fisher Scientific, catalog number: 353047)
6. Greiner 6-well cell culture plate (Fisher Scientific, catalog number: 657160)
7. Heathrow Scientific slide mailer (Sigma-Aldrich, catalog number: HS120557)
8. Fisherbrand glass Pasteur pipette (Fisher Scientific, catalog number: 13-678-20A)
9. USA Scientific SealRite 0.5 mL microcentrifuge tubes (USA Scientific, catalog number: 1605-0000)
10. USA Scientific SealRite 1.5 mL microcentrifuge tubes (USA Scientific, catalog number: 1615-5510)
11. Ted Pella sable brush #0, 1.3 mm W × 8.0 mm L (Ted Pella, catalog number: 11810)
12. Ted Pella sable brush #1, 1.5 mm W × 9.5 mm L (Ted Pella, catalog number: 11812)

## Equipment

1. Titer plate shaker model 4625 (Lab-line Instruments, catalog number: 0101-1383)
2. Heratherm incubator (Fisher Scientific, catalog number: 50125590)
3. Fisherbrand water bath (Fisher Scientific, catalog number: 15-462-2Q)
4. Stereo microscope (Leica, model: MZFLIII)
5. Confocal microscope (Zeiss, model: LSM 710)

## Software and datasets

1. Zen v2.3 black edition (Zeiss), https://www.micro-shop.zeiss.com/en/us/softwarefinder/software-categories/zen-black/
2. Prism 10 for Windows 64-bit (GraphPad, version 10.2.3 9403, April 21, 2024)
3. Fiji ImageJ, open source, https://www.nature.com/articles/nmeth.2019)
4. Adobe Photoshop 2023 Adobe Photoshop Version: 24.7.0 20230719.r.643 efe3886 ×64, September 2023)

## Procedure

- **Prior to expansion procedure: fixation, dissection, and immunostaining**
  1. Fixation
    - Prior to the expansion procedure, fix 5-days-post-fertilization (dpf) larvae in 2% TCA solution for 3 h at room temperature (RT) on a rocker.
    - Remove the 2% TCA solution and wash larvae with 1× PBS three times.
  2. Dissection
    - Pin larvae down to a Sylgard-coated dish and remove the eyes and skin on top of the head of the larvae with forceps.
    - Similarly, remove the notochord with forceps.
    - Remove the head of the larvae by making a cut at the level of the yolk sac.
    - Place the dissected brain into a 0.5 mL microcentrifuge tube with 350 μL of blocking solution (for more details, see Figure 1).
  3. Immunostaining
    - Incubate dissected heads in the blocking solution for 3 h at RT on a rocker.
    - Remove the blocking solution, add appropriately diluted primary antibody in 300 μL of blocking solution, and incubate the samples overnight at RT on a rocker.
    - Remove the primary antibody solution and rinse with 0.5% PBS-Trx three times.
    - Wash samples in 0.5% PBS-Trx for 10 min, 15 min, and 30 min with fresh solution changes each time, on a rocker.
    - Remove 0.5% PBS-Trx and add appropriately diluted secondary antibody in 300 μL of blocking solution. Incubate the samples for 4 h at RT in the dark on a rocker.
    - Remove the secondary antibody solution and rinse with 1× PBS three times.
    - Wash samples in 1× PBS four times for 15 min on a rocker.
    - Proceed with the following protocol (expansion steps) on the same day.
*Note: All incubations and washes take place on a rocker.*
*Note: Some fluorescent dyes conjugated to antibodies are not compatible with the expansion protocol and will degrade, especially those in the cyanine family (Cy3, Cy5, and Alexa 647) [1,13]. Recommended secondary antibodies include CF 405M, Alexa 488, Alexa 546, Alexa 594, CF 633, and Atto 647N. Please refer to the Reagents section for a list of fluorescent antibodies that have been successfully used in this protocol [1].*
    **Figure 1.**
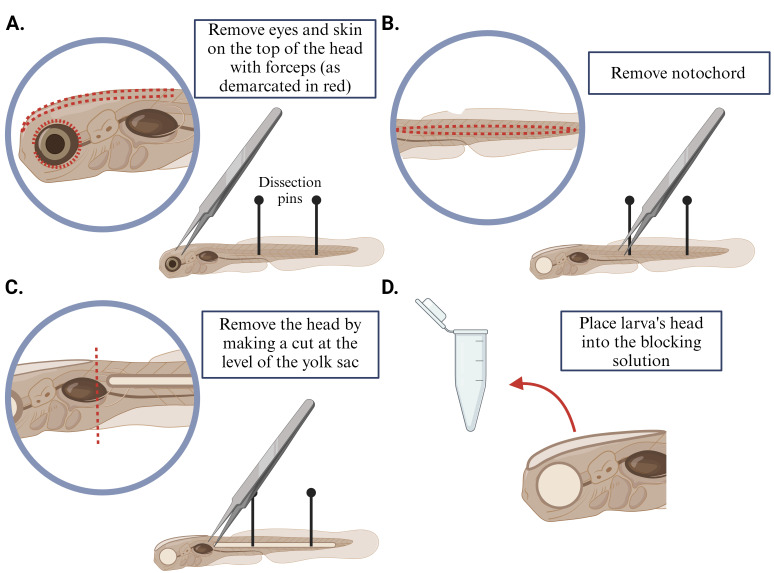
Brain dissection of larval zebrafish
- **Day 1: anchoring**
*Note: Stained samples should be protected from light as much as possible throughout the remainder of the procedure to prevent bleaching of the fluorophores.*
  1. Following staining, carefully slide the tips of the forceps beneath the larva’s skin and remove the skin from the brain.
  2. Individually place the brains into a 24-well plate using a glass pipette and add 200 μL of anchoring solution to each well.
  3. Place the plate on a shaker overnight (for at least 16 h) at RT to enhance the diffusion of the anchoring solution through the sample.
*Note: It is crucial to set the shaker at an appropriate speed (40–70 rpm) to prevent the brains from sticking to the walls of the well, as this may lead to them drying out and/or fail to allow the anchoring solution to penetrate the samples deep enough for the expansion process to work effectively.*
- **Day 2: gel polymerization**
  1. After the overnight incubation, remove the anchoring solution from each well and wash samples with 200 μL of 1× PBS. This step should be performed on a shaker (at speed 40–70 rpm) for 10 min at RT and repeated twice.
  2. While the samples are washing, construct custom-made coverslip wells for use during gel polymerization: Use a microscope slide as a base, place two coverslips (22 × 22), one on either end of the slide, and add ~2 μL of water to adhere the coverslips to the slide. Apply a second set of coverslips over the first set using super glue. Make enough coverslip wells for every sample. (For more details, see Figure 2A. For a video demonstration, see Tillberg et al. [1].)
*Note: Two sets of coverslips are used to accommodate for the thickness of the sample (~300 μm). Use as many coverslips as needed to avoid crushing the sample.*
    **Figure 2.**
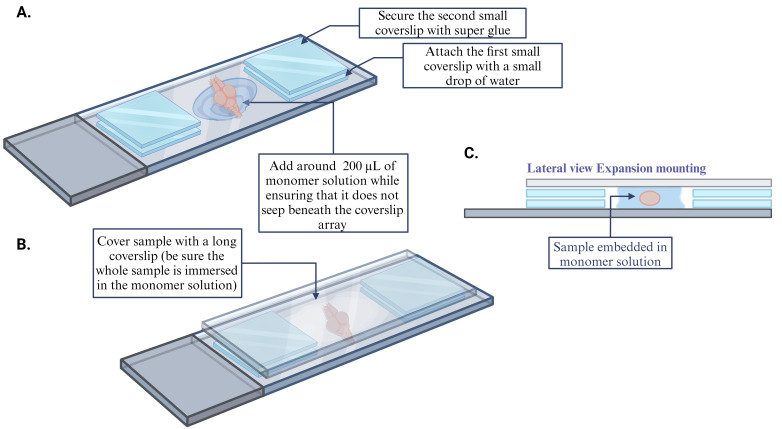
Sample mounting for gel polymerization
  3. After washing with 1× PBS, remove the 1× PBS from each well and replace it with 376 μL of monomer solution, and then sequentially add 8 μL of 0.5% 4-HT stock solution.
  4. Rock the plate on a shaker (at speed 40–70 rpm) for 10 min at RT, allowing the 376 μL of monomer solution to mix with the 8 μL of 0.5% 4-HT stock solution and to allow the mixture to diffuse into the sample.
  5. Remove the solution from step C4 and add 376 μL of fresh monomer solution. Then, add 8 μL of 0.5% 4-HT stock solution.
  6. This time, rock the plate on a shaker (at speed 40–70 rpm) for 5 min at RT, again allowing the 376 μL of monomer solution to mix with the 8 μL of 0.5% 4-HT stock solution and to allow the mixture to diffuse into the sample.
*Note: Steps C7–C9 should be performed on one sample at a time and executed quickly to prevent premature polymerization of samples. Once step C9 is performed on one sample, go back to step C7 and repeat with each sample.*
  7. Add 8 μL of 10% TEMED stock solution and rock the plate on a shaker (at speed 40–70 rpm) for 1 min.
  8. Add 8 μL of 10% APS stock solution and rock the plate on a shaker (at speed 40–70 rpm) for 30 s. Together, these reagents form the gelling solution and initiate polymerization.
  9. Before the polymerization is completed, transfer the samples using a glass Pasteur pipette with a small amount of gelling solution (~100 μL) to the custom-made coverslip wells. Mount the sample either dorsal or ventral side up, ensuring that there are no air bubbles, and place a coverslip over the sample (24 × 50) (for more details, see Figure 2B).
*Note: Make sure that enough solution is added along with the sample to the coverslip well. Too much could disturb the structure of the well, and too little could dry out the sample.*
  10. Place the samples into a slide mailer and incubate them at 4 °C for 50 min. This will help to reduce the polymerization rate of the gel and ensure proper diffusion of the solution into the brain [15,16].
*Note: Handle the samples with care while placing them in a slide mailer, taking care not to move the coverslip on top of the sample, as this will shift the mounting orientation.*
  11. Move the samples into a 37 °C incubator for 2 h, resulting in gel formation in and around the brain [15,16].
  12. While the samples undergo incubation, add 250 μL of proteinase K solution (8 U/mL) to 1.5 mL microcentrifuge tubes (one tube per sample).
  13. Once the incubation period is complete, remove the coverslips (both 22 × 22 and 24 × 50) from the mounted sample under a dissecting microscope.
  14. Moisten the top of the sample (now in the hydrogel) with the digestion buffer using a paintbrush to ensure that it remains hydrated, and carefully cut the gel surrounding the sample using a scalpel or razor blade (for more details, see Figure 3).
*Note: Introduce an asymmetrical cut to the gel to establish the orientation of the sample (ventral or dorsal) for reference later. Due to TCA fixation, pigment from the skin over the head remains on the dorsal side of the brain, making it easy to identify the orientation.*
    **Figure 3.**
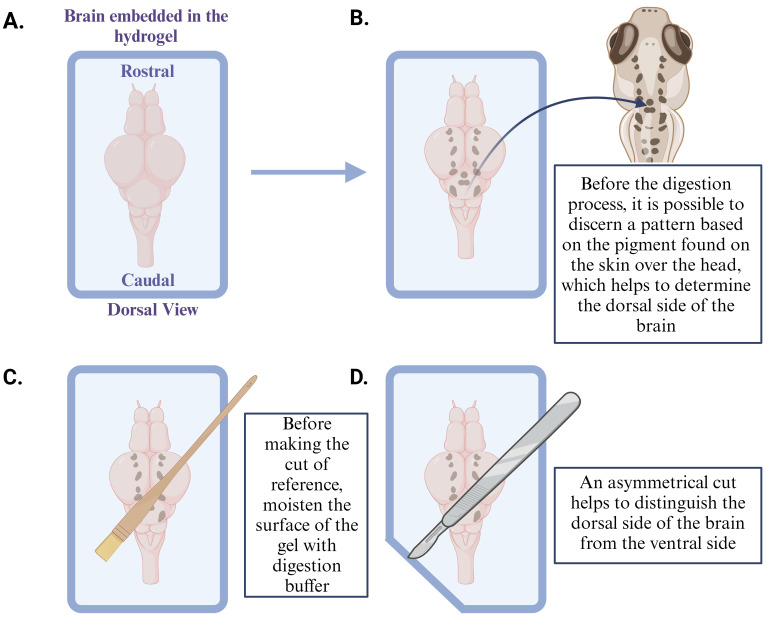
Identify the orientation of the sample (dorsal side here) embedded in the hydrogel
  15. Using a paint brush, transfer the samples into the microcentrifuge tubes containing the proteinase K solution (8 U/mL).
  16. Incubate the samples overnight for 12 h in a water bath at 50 °C. This step will allow proteinase K to digest the brain tissue, resulting in a translucent sample.
- **Day 3: expansion**
  1. Following the overnight incubation, transfer the samples into a 6-well plate using a paintbrush, with one sample per well.
  2. Remove any remaining digestion buffer.
  3. Wash the samples with 5 mL of cell culture–grade water for 30 min on a shaker. To induce gel expansion, perform four additional washes, each one for 30 min to allow the tissue to gradually expand.
*Note: If imaging samples on a different day, do not wash with water; instead, store samples in 5 mL of 1× PBS at 4 °C in the dark, until you are ready to image.*
  4. While the gel is expanding, prepare the microscope slides for imaging (one slide per sample): start by cleaning the slides with 50 μL of water in a laminar fume hood and dry with Kimwipes. Repeat this twice. Next, clean slides with 50 μL of 70% ethanol and dry with Kimwipes. After the ethanol has evaporated, apply 50 μL of Poly-D-Lysine (1 mg/mL) to the center of the slide and incubate at RT, in the fume hood, for 30 min. Subsequently, remove any excess Poly-D-Lysine and perform three washes with 50 μL of cell culture–grade water and let slides dry before mounting sample (for more details, see Figure 4).
*Note: It is essential to mark the reference point of Poly-D-Lysine placement on the slide, as that will serve as the designated spot for mounting the sample.*
  5. After the washes are done and the expansion process is complete, carefully remove all the water from the well (for more details, see Figure 5A).
*Note: Only work with one sample at a time. Leave water in the wells of the samples you are not actively working with to ensure that they remain hydrated.*
  6. Dry the bottom of the well with a Kimwipe carefully to avoid touching the gel.
  7. Place a coverslip in the well and gently slide the sample over the coverslip using a paintbrush. Ensure that the orientation for mounting is ventral side up in an upright confocal microscope. This ensures the Mauthner cell is easily accessible, as it is located on the ventral side of the brainstem (for more details, see Figure 5B)
*Note: This method ensures that the sample is not directly manipulated, thereby reducing the risk of breaking the gel.*
  8. Using forceps, carefully remove the coverslip with the gel and slide the gel onto the microscope slide with Poly-D-Lysine using a paint brush (for more details, see Figure 4 and 5C).
    **Figure 4.**
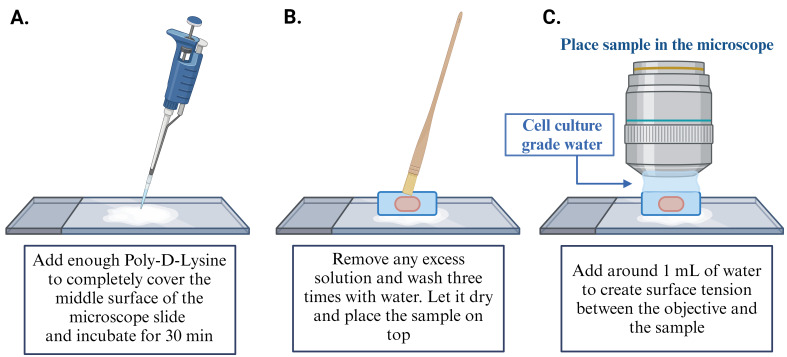
Mount the gel and image
  9. To locate the brain, specifically the Mauthner cell within the gel, use a dissecting scope and place a small piece of Kimwipe (~3 mm × 3 mm) on top of the gel, around the hindbrain region, to serve as a reference point for the general vicinity of the Mauthner cell.
*Note: Although the tissue has been digested, it is still possible to determine the original location and orientation of the brain due to the presence of residual pigment on the dorsal side of the brain, an effect of TCA fixation (for more details, see Figure 3B).*
  10. To find the sample using an upright confocal microscope, use a 10× objective with the transmitted light on and identify the position of the Kimwipe.
  11. Carefully remove the Kimwipe with forceps and add ~1 mL of cell culture–grade water to maintain gel hydration and assist in visualizing the sample using a 40× water immersion objective.
  12. Locate the lateral dendrite of the Mauthner cell and begin imaging (for more details, see Figure 4C).
    **Figure 5.**
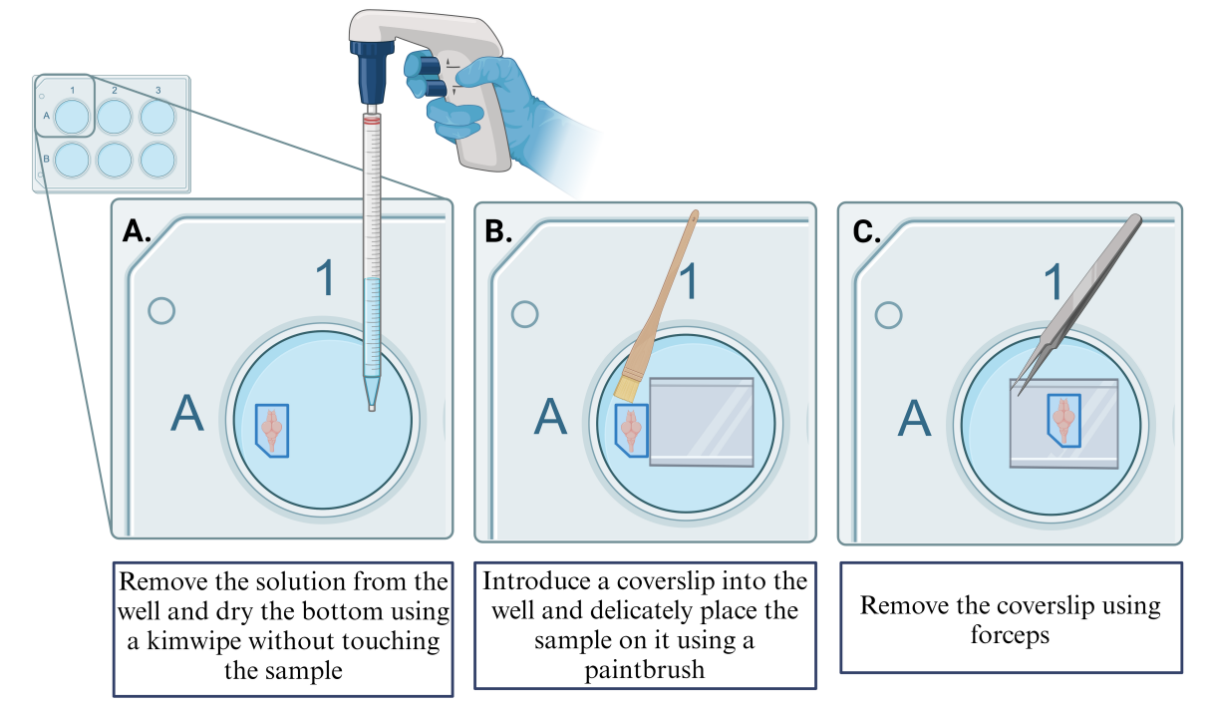
Remove sample from the 6 well-plate

## Data analysis

Images were acquired using the Zeiss LSM 710 confocal microscope with a 40× (1.0 NA) water immersion objective. The acquired images of expanded samples, when compared to non-expanded samples, retained normal anatomical features (Figure 6, 10). Data analysis methods, which have been described in Cárdenas-García et al., 2024 (Results and Methods, 10), were used to determine the achieved expansion factor and colocalization, distribution, and occupancy of synaptic components at CEs. To give a brief overview of these methods, labeling of the oval CE contact areas on the Mauthner cells was measured pre- and post-expansion in Fiji. These measurements resulted in a ~3.9× lateral expansion factor and a ~13× area expansion factor (10). Colocalization of components was determined by analyzing fluorescence overlap and was performed in Fiji, with the JACoP plugin, followed by quantification using the Manders’ coefficient. Differential distribution of molecular components was also determined in Fiji by defining regions of interest (ROIs) in different areas of the contact and quantifying the fluorescence intensity in each ROI. Additionally, pre- vs. post-synaptic distribution of electrical components was more easily discernible given the increase in resolution in post-expanded samples and was found using line scan in Fiji. Similarly, line scan analysis was used to determine the distribution of fluorescence of any component relative to another. Finally, all this data was combined to determine the organization and proportion of these synaptic proteins at CE contacts (please see Cárdenas-García et al. [11] for more details). Finally, while this protocol proved to be very efficient, its main limitation is that it results in some loss of fluorescence intensity due to the necessary use of protease, which allows for isotropic expansion.

## Validation of protocol

This protocol or parts of it has been used and validated in the following research article:

- Cárdenas-García et al. (2024). The components of an electrical synapse as revealed by expansion microscopy of a single synaptic contact. eLife (Figure 1, panel E; Figure 2, panel A–C; Figure 3, panel A–D; Figure 4, panel C–E; Figure 5, panel A–C; Figure 6, panel C, E, G, H).
